# A Pan-Draft Metabolic Model Reflects Evolutionary Diversity across 332 Yeast Species

**DOI:** 10.3390/biom12111632

**Published:** 2022-11-03

**Authors:** Hongzhong Lu, Eduard J. Kerkhoven, Jens Nielsen

**Affiliations:** 1 State Key Laboratory of Microbial Metabolism, School of Life Sciences and Biotechnology, Shanghai Jiao Tong University, Shanghai 200240, China; 2 Department of Biology and Biological Engineering, Chalmers University of Technology, Kemivägen 10, SE412 96 Gothenburg, Sweden; 3 BioInnovation Institute, Ole Måløes Vej, DK2200 Copenhagen, Denmark

**Keywords:** yeast species, draft metabolic model, metabolic evolution, trait diversity

## Abstract

Yeasts are increasingly employed in synthetic biology as chassis strains, including conventional and non-conventional species. It is still unclear how genomic evolution determines metabolic diversity among various yeast species and strains. In this study, we constructed draft GEMs for 332 yeast species using two alternative procedures from the toolbox RAVEN v 2.0. We found that draft GEMs could reflect the difference in yeast metabolic potentials, and therefore, could be utilized to probe the evolutionary trend of metabolism among 332 yeast species. We created a pan-draft metabolic model to account for the metabolic capacity of every sequenced yeast species by merging all draft GEMs. Further analysis showed that the pan-reactome of yeast has a “closed” property, which confirmed the great conservatism that exists in yeast metabolic evolution. Lastly, the quantitative correlations among trait similarity, evolutionary distances, genotype, and model similarity were thoroughly investigated. The results suggest that the evolutionary distance and genotype, to some extent, determine model similarity, but not trait similarity, indicating that multiple mechanisms shape yeast trait evolution. A large-scale reconstruction and integrative analysis of yeast draft GEMs would be a valuable resource to probe the evolutionary mechanism behind yeast trait variety and to further refine the existing yeast species-specific GEMs for the community.

## 1. Introduction

It is known that there are over 1500 different yeast species on earth with diverse metabolic functions, which are currently widely exploited in various fields including basic biology study, industrial biotechnology, ecology, etc. [1,2,3]. In general, yeast species can be classified as conventional or non-conventional yeasts [4]. Conventional yeasts including *Saccharomyces cerevisiae* and *Schizosaccharomyces pombe* are regarded as eukaryotic model microbes, while non-conventional yeasts contain dozens of useful yeast strains, including *Yarrowia lipolytica*, *Pichia pastoris*, and *Kluyveromyces marxianus*. As compared with conventional yeasts, non-conventional yeasts harbor some unique metabolic advantages, such as high-density aerobic fermentation, extremely diverse metabolic function, wide substrate utilization spectrum, and strong stress resistance [5,6]. Therefore, these yeasts are widely used in the production of organic acids, sugar alcohols, terpenoids, lipids, enzymes, etc. [7,8]. Because of the quick development in sequencing and bioinformatic tools, genome sequences and detailed gene function annotations for 332 yeasts are now available to the community [1], laying the groundwork for further exploitation and application of genomics in the study of yeast biology, evolution, and biotechnology [9]. However, it has become important to reconstruct genome-scale metabolic models and to derive pan-metabolic models for all sequenced yeast species [1] in order to understand yeast metabolic evolution holistically.

Genome-scale metabolic models (GEMs), as one of the most important classes of computational models, are important for probing the complex genotype–phenotype relationships [10]. Until now, there have been different procedures and toolboxes to automatically build large-scale GEMs [11,12,13,14]. In general, these procedures can be classified as model construction from a simplified template model and from an open-source biochemical database. For example, CarveMe can generate GEMs for bacteria based on a template model built from the BiGG reaction database [12]. By contrast, the RAVEN toolbox provides two alternative methods to build draft GEMs from two popular databases, i.e., MetaCyc and KEGG, by blasting the queried genome against a comprehensive gene/protein sequence database [14]. Those existing toolboxes provide a convenient way to build GEMs for any microbe with a whole sequenced genome available [15], although the quality and prediction performance of GEMs could be influenced by many factors, such as the types of gap-filling algorithms and the choice of the template models. By comparison, it shows that the RAVEN toolbox performs better to reflect the metabolic diversity for less-studied species [15].

Until now, various procedures have been used to build GEMs for yeast species [16], among which, Lu et al. built GEMs for 332 yeast species using semi-automated procedures (the related models were renamed as semi-auto GEMs) [17]. In this procedure, a template model from *S. cerevisiae* GEMs, Yeast8 was used and additional reactions were added by re-annotating the pan-genome to generate a so-called simplified template model. Next, with this template model, the semi-auto GEMs for the studied yeast species were generated automatically based on the ortholog gene relations, followed by multiple rounds of manual curation to improve the model prediction performances. By comparison, the quality and scope of semi-auto GEMs could be comparable with those of existing published yeast species-specific GEMs. In addition, Correia et al. built a consensus pan-GENRE for fungi by combining the gene annotation from several popular fungal strains, including *S. cerevisiae* and *S. pombe*, which were then used to build GEMs for 33 yeasts/fungi [18]. As an alternative procedure, the most recent *S. cerevisiae* GEMs, Yeast8, was widely used as the template model for the GEMs reconstruction of single species, and the bi-directional blast was employed to infer the existence of reactions in other yeast species [16], such as *Y. lipolytica* [19] and *K. marxianus* [20]. Thus, the reconstruction of GEMs for yeast species relied heavily on Yeast8 [21], which, to some extent, may ignore the metabolic diversity encoded in the genomes of other numerous non-conventional yeast species.

In this study, to further encompass yeast metabolic diversity and to explore yeast metabolic evolution, draft GEMs for each sequenced yeast species (draft GEMs) were first automatically built using the RAVEN toolbox. Then, with standard annotation of reaction IDs from different sources, a pan-draft metabolic model was compiled as the knowledge base for the most sequenced yeast species to date. Next, these draft GEMs and pan-draft models were used to explore the tendencies in genomic and metabolic evolution, as well as the potential correlations among model similarity, trait similarity, genotype similarity, and phylogenetic evolutionary distance across yeast species. Additionally, the model similarity calculated based on draft GEMs and semi-auto GEMs was further compared to display the advantage of draft GEMs in reflecting the diversity of yeast metabolism. The systematic reconstruction of yeast draft GEMs and pan-draft metabolic model paves the way to further refine gene function annotation for all yeast species, and therefore, will set a solid basis to enhance the quality of different yeast species-specific GEMs in the coming future.

## 2. Materials and Methods

### 2.1. Collection of Proteomes for 332 Yeast Species

All the yeast genomes and proteomes were downloaded from https://figshare.com/articles/Tempo_and_mode_of_genome_evolution_in_the_budding_yeast_subphylum/5854692, accessed on 23 October 2018) [1]. The protein ID conversion was conducted based on the ortholog ID, gene ID, and protein ID mapping, which was queried from [1]. In each proteome, the protein ID was started with the yeast species name, such as “*Yarrowia_lipolytica*@Seq_6369”. This type of protein ID and the corresponding protein sequence were used in the following work.

### 2.2. Reconstruction of Draft GEMs Using the RAVEN Toolbox

The current RAVEN v2 [14] provides two procedures (getKEGGModelForOrganism and getMetaCycModelForOrganism) for reconstruction of draft GEMs based on the input proteome. The main parameters used in the method getMetaCycModelForOrganism (RAVEN ”MetaCyc”) are percent identity and bit-score. Since these parameters in sequence blast analysis could affect model quality, different values of these two parameters were explored to estimate the best combinations. In this step, the detailed gene function annotation of *S. cerevisiae* (containing the reaction information) from MetaCyc [22] was downloaded as the reference to evaluate the effects of percent identity and bit-score. Here, the accuracy was defined as the following formula:

(1) $$Model\;accuracy=\frac{TP+TN}{TP+TN+FP+FN}$$

where TP, TN, FP, and FN represent true positive, true negative, false positive, and false negative, respectively, when comparing the output of the RAVEN ”MetaCyc” procedure with the reference model of *S. cerevisiae* that already existed in MetaCyc database.

According to the above analysis, the percent identity and bit-score were set as 55% and 110, respectively, for the RAVEN ”MetaCyc” procedure. Since the draft GEMs reconstruction using the procedure getKEGGModelForOrganism (RAVEN ”KEGG”) need map protein homology to the ortholog gene families in KEGG, the pretrained HMMs (euk90_kegg100) and the default parameters were used to build draft GEMs for all yeast species.

### 2.3. Reconstruction of a Pan-Draft Metabolic Model for 332 Yeast Species

For the reconstruction of comprehensive pan-draft metabolic models for all yeast species, the draft GEMs for each yeast from the two procedures of the RAVEN toolbox were combined. First, the reaction ID from each of the two complementary models was standardized by querying the corresponding ID from known biochemical reaction databases, such as MetaNet [23] and modelSeed [24] databases. Then, the unique ID from MetaNet was used for each reaction if possible. If a MetaNet ID could not be found, the original ID from the MetaCyc or KEGG database was directly used. After this annotation, a combined reaction ID list and the corresponding reaction information (including the reaction formula and gene association) were summarized for each yeast species. Meanwhile, to obtain the subpathway information for each reaction, the KEGG ID was queried utilizing ID mapping. Based on the KEGG ID of each reaction in the pan-draft model, the subsystem definition from the KEGG database was assigned for each reaction. Subsequently, all the above unique reaction IDs were compiled to formulate into the pan-draft model of 332 yeast species. To analyze the effect of the number of yeast species on the size of the pan-draft model, the yeast species were randomly selected to calculate the number of pan reactions (all reactions contained in the sampled yeast species), core reactions (existing in each of sampled yeast species), and accessory reactions (existing only in part of sampled species).

### 2.4. Models’ Similarity Calculation

For the metabolic model similarity analysis, the reaction IDs from two GEMs were extracted and compared. The model similarity for any two yeast species was represented by the Jaccard distance based on reaction existence in each metabolic model. The Jaccard distance was calculated based on the following formula:

(2) $$Jaccard\;similarity=\frac{R_{A}⋂R_{B}}{R_{A}\cup R_{B}\;}$$

where R_A_ and R_B_ represent the metabolic reactions in yeast species A and B, respectively.

### 2.5. Trait Similarity Calculation

In order to evaluate the trait similarity for any two yeast species, the physiology datasets including the growth condition on various carbon and nitrogen sources were downloaded from two previous studies [1,25]. These physiology datasets were also compiled in our previous work [17]. In summary, 329 of 332 yeast species have detailed usage profiles across 32 different substrates. Then, these physiology datasets were used to infer the trait similarity based on the above procedure in the calculation of the Jaccard distance.

### 2.6. Evolutionary Distance Calculation across Yeast Species

The time-calibrated phylogenetic tree for 332 yeast species was downloaded from https://figshare.com/articles/Tempo_and_mode_of_genome_evolution_in_the_budding_yeast_ subphylum/5854692, accessed on 23 October 2018) [1]. Next, the R function-cophenetic from R package ape v 5.0 (http://ape-package.ird.fr/) was used to calculate the pairwise evolutionary distance across yeast species.

### 2.7. Genotype Similarity Calculation

It is shown that different combinations of two yeast species could have the same evolutionary distance. Thus, to further characterize yeast genomic evolution in detail, the gene ortholog relationships from [1] were employed to estimate the existence of each gene across yeast species. In this analysis, the unified ortholog IDs for each yeast were collected based on the mapping between the ortholog IDs and gene IDs from [1]. Then, the genotype similarity for any two yeast species was calculated in a similar way using the Jaccard distance based on the existence of ortholog IDs across yeast species.

### 2.8. Statistical Analysis

For two group comparisons in this work, a two-tailed Wilcoxon rank sum test was conducted.

## 3. Results

### 3.1. Reconstruction of Draft GEMs Using the Latest RAVEN Toolbox

**Figure 1:**
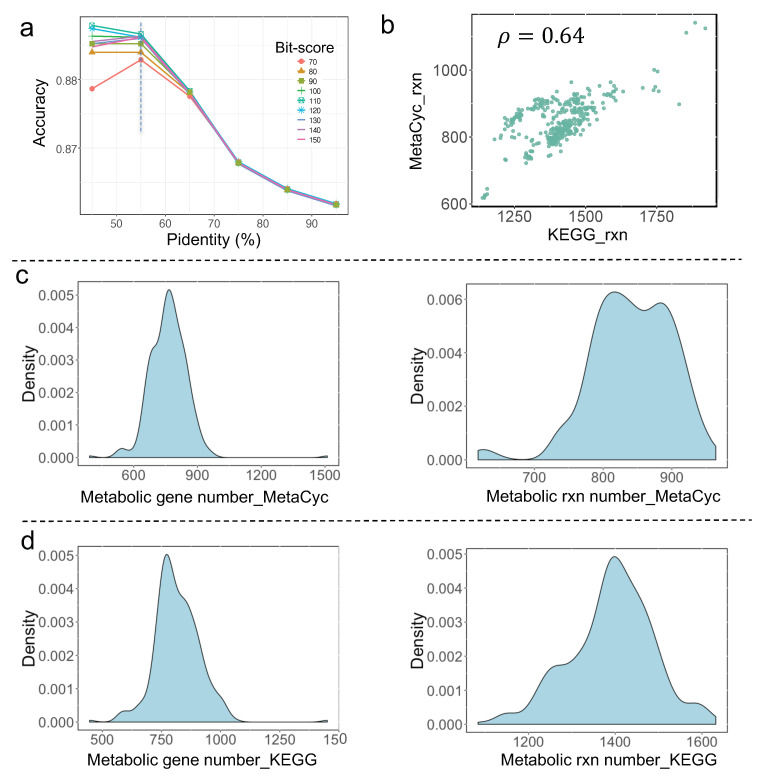
Draft yeast species-specific metabolic model reconstruction using the RAVEN toolbox: (**a**) Effects of the parameters’ cut-offs on model quality (defined as accuracy) from the RAVEN getMetaCycModelForOrganism procedure. Here, the existing model information from BioCyc (as part of MetaCyc) for *S. cerevisiae* S288c was set as the reference during the comparison; (**b**) correlation in total reaction number for draft GEMs from RAVEN getMetaCycModelForOrganism and from RAVEN getKEGGModelForOrganism procedures, 𝜌 is Pearson’s correlation coefficient; (**c**) distribution of metabolic gene and reaction number from 332 yeast species draft GEMs reconstructed using RAVEN getMetaCycModelForOrganism; (**d**) distribution of metabolic gene and reaction number from 332 yeast species draft models reconstructed using RAVEN getKEGGModelForOrganism.

Yeast metabolism has undergone systematic evolution to adapt to the growth niches. To encompass the metabolic diversity of yeast species as much as possible, two alternative procedures were employed in the RAVEN toolbox [14], that is, built draft GEMs based on MetaCyc and KEGG databases, respectively, were adopted in this study. Different from our previous work where a template model, Yeast8 [17], was used, two more comprehensive databases, i.e., MetaCyc and KEGG, used in RAVEN could largely expand the size of the referred biochemical reaction network, thus, to some extent, helping to reduce the metabolic model similarity between any two yeast species. Firstly, as the RAVEN ”MetaCyc” procedure relied heavily on the sequence blast analysis, two main pivotal parameters, percent identity (Pidentity) and bit-score, were systematically evaluated before the automatic reconstruction of draft GEMs for yeast (Figure 1a, Material and Methods). Next, all the yeast proteome datasets were downloaded from [1] and used as input to generate draft GEMs for 332 yeast species plus 11 outgroup fungal species, which then laid a solid foundation for the reconstruction of pan-draft metabolic models for sequenced yeast species to date.

### 3.2. Comparative Analysis of All Draft Metabolic Models

At first glance, the number of reactions in draft GEMs produced by the RAVEN “KEGG” and RAVEN “MetaCyc” procedures, respectively, are positively correlated (Figure 1b). The detailed distribution of gene number and reaction number from these draft GEMs are shown in Figure 1c,d. In general, the gene number is typically in the range of 500–1000, and the reaction number is in the range of 600–1000, for yeast draft GEMs from the RAVEN “MetaCyc” procedure. By contrast, the gene number is in the range of 600–1400, and the reaction number is in the range of 1100–1600, for yeast draft GEMs from the RAVEN “KEGG” procedure. It should be noted that only reactions with gene associations were kept for all draft GEMs created by RAVEN, and, the compartment information for each reaction was left out. Thus, the reaction number in these draft GEMs was considerably less than that of the manually calibrated yeast GEMs [17], while it is found that draft GEMs encompass specific reactions which are absent in those manually calibrated GEMs. For example, during the update of consensus yeast GEMs for *S. cerevisiae* from Yeast8 to Yeast9, over 100 reactions can be found from draft GEMs which will be added into Yeast8 to generate Yeast9 (unpublished datasets).

**Figure 2:**
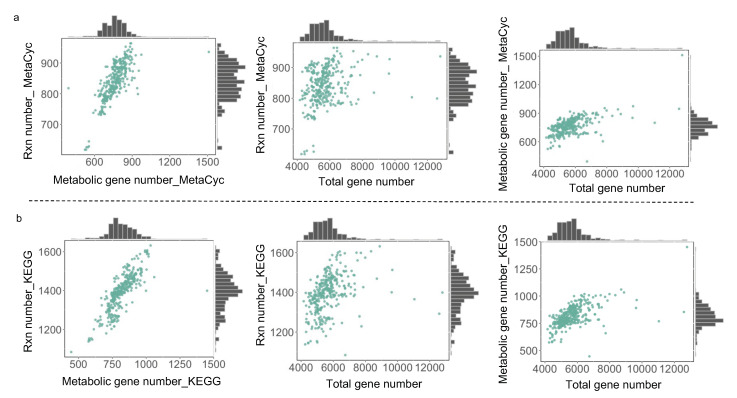
Total gene number determines the metabolic reaction and gene number for draft GEMs from RAVEN toolbox. Correlations among metabolic gene, reaction number, and total gene number from 332 yeast species draft models reconstructed using RAVEN getMetaCycModelForOrganism (**a**) and RAVEN getKEGGModelForOrganism (**b**).

For those draft GEMs from the aforementioned two procedures, the correlation between the reaction number and gene number across yeast species exhibits a similar pattern (Figure 2a,b), suggesting that the number of metabolic genes encoded in the yeast genome determines the total number of reactions in draft GEMs. When further assessing the effects of gene set on the size of draft GEMs, it is observed that both the reaction number and the gene number were not always increased along with the expansion of the gene set. In detail, if the total number of genes is between 4000 and 8000, it appears that the number of reactions increases along with the size of the gene set. However, once the overall number of genes surpasses 8000, the numbers of genes and reactions contained in draft GEMs reach a plateau, even decreasing at certain data points. As a result, the draft GEM reconstruction together with total gene number analysis initially indicated that there may be a potential upper limit on the number of metabolic reactions at the single species level for the models created directly by RAVEN v 2.0 without manual curation.

### 3.3. Pan-Draft Metabolic Model Reconstruction and Analysis for Budding Yeasts

**Figure 3:**
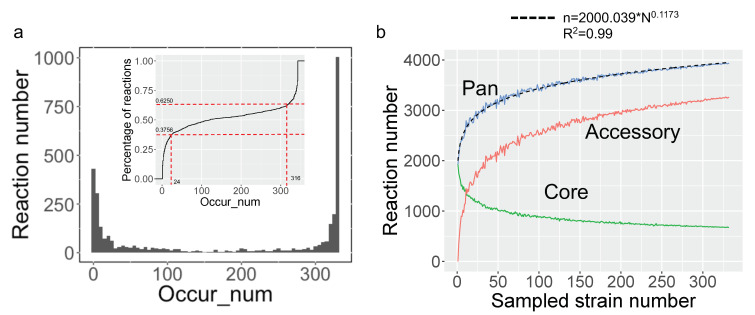
Statistical analysis of the pan-draft metabolic models: (**a**) Distribution of reactions based on their occurrence number in 332 yeast species plus 11 outgroup fungal species. The small graph in (**a**) represents the cumulative density of reactions based on its existence across 332 yeast species plus 11 outgroup fungal species; (**b**) profiles of pan-, core, and accessory reactions along with the number of sampled yeast species. The dotted line represents the predicted pan-reaction number from the Heaps’ law.

To characterize the metabolic diversity encoded in the pan-genome of 332 yeast species, a pan-draft metabolic model was subsequently created by merging all reactions and genes from the aforementioned draft GEMs. The standardization of reaction IDs was conducted to eliminate repetitions in reactions across all draft GEMs. There are about 3940 distinct reactions in yeast pan-draft metabolic models as a whole. The occurrence number of each reaction across yeast species was calculated. Similar to the pan-gene occurrence in previous studies [26], we discovered that all reactions could be divided into three major types when taking into consideration their existence in all yeast species, with 37.5% of reactions existing in over 316 species, 37.56% of reactions existing in less 24 species, and 25.94% of reactions existing in 24–316 species (Figure 3a). This investigation demonstrated that the expansion of the yeast pan-metabolic model was significantly facilitated by species-specific reactions.

Based on the reaction occurrence frequency in the species under study, the pan-, accessory, and core reactions can be further defined in the so-called pan-reactome, which encompasses all related reactions. All reactions existing in pan-reactome are defined as pan-reactions. The reactions that are common to all yeast species are categorized as core reactions, while the reactions that only exist in some yeast species are categorized as accessory reactions. To evaluate the impact of the number of sampled species on the composition of the pan-reactome, the number of pan-, accessory, and core reactions were calculated when the number of sampled species was gradually increased (Figure 3b). It shows that the counts of pan- and accessory reactions increased swiftly at the start of sampling until the number of randomly selected species was over 50. It has been reported that Heaps’ law could be applied to assign the ”close” or ”open” property to a pan-genome for a group of species when more genomes were regularly added [27]. Using the same premise, the Heaps’ law was used to describe the characteristics of the pan-reactome for the yeast species. The fitting formula of Heaps’ law in this study is *n* = 2000.039*N^0.1173^, where N is the number of sampled yeast species and *n* is the total number of reactions. According to the definition of Heaps’ law, the index in the above formula is significantly smaller than one, demonstrating that the pan-reactome of yeast species has a “closed” property. Thus, it is speculated that the yeast pan-reactome will tend to be stable, even when adding more newly sequenced yeast species. 

**Figure 4:**
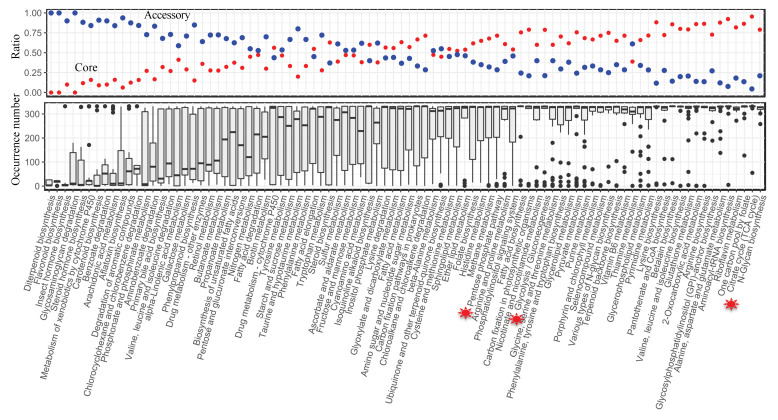
Analysis of the pan-draft metabolic model for 332 yeast species at the subsystem level. The subsystem definitions are from the KEGG database. The upper graph summarizes the ratio of core and accessory reactions in each subsystem and the lower graph summarizes the occurrence number of all reactions from each subsystem across 332 yeast species. The red and blue circle in upper graph dot represent the ratio of core and accessory reactions in each subsystem, respectively. The red symbols in the bottom graph represents the core metabolic pathways in yeast species, inclusing TCA cycle, EMP and PPP pathways.

The composition of the pan-reactome was further explored at the subsystem level. Within the pan-model, the subsystem definition based on KO annotation from the KEGG [28] database was given for each reaction. Subsequently, the ratio of core and accessory reactions in each subsystem was computed, and then the general distribution of reaction occurrence was compared at the subsystem level. Comparatively, we discovered that the majority of core reactions were present in the subsystems involved in core metabolic processes, such as the TCA cycle and amino acid biosynthesis, whereas accessory reactions predominated in the subsystems involved in secondary metabolism (Figure 4). It is noted that, for some ancient pathways, taking the PPP pathway as an example, 25% of the pan-reactome belongs to the accessory reactions, indicating that based on the current definition of a subsystem from the KEGG database, a part of the reactions from the PPP pathway was still variable across yeast species.

### 3.4. Correlations among Trait Similarity, Model Similarity, and Evolutionary Distance

With long-term evolution, strains always gain or lose a trait to ensure greater fitness in their environment. It is always appealing to illustrate intricate relations between genotypes and phenotypes for distinct yeasts [3,29]. Here, we employed draft GEMs comparative analysis, together with a correlation analysis of genotype, phylogeny evolutionary distance, and phenotype, to investigate the probable mechanism driving yeast metabolic diversity.

**Figure 5:**
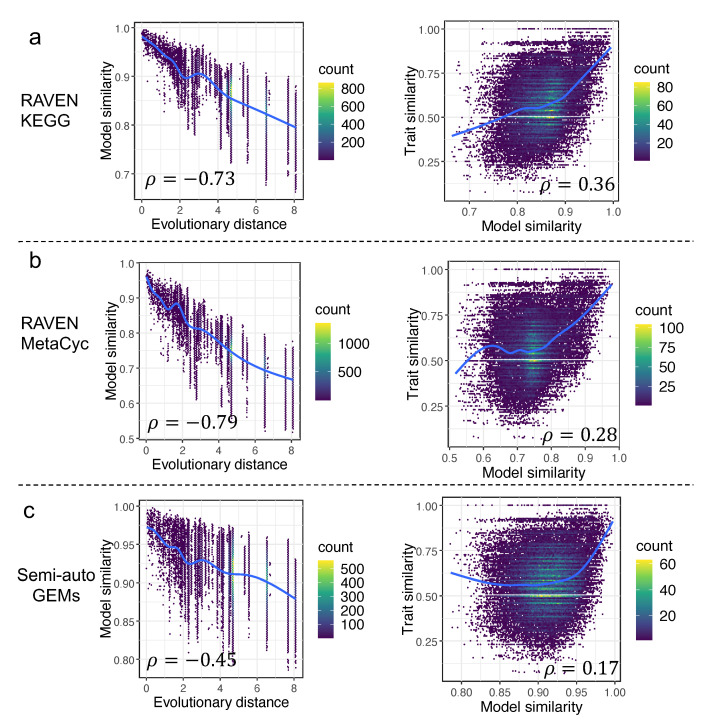
Correlation analyses among model similarity, evolutionary distance, and trait similarity across 332 yeast species for draft GEMs built using RAVEN getKEGGModelForOrganism (**a**), getMetaCycModelForOrganism (**b**), and the semi-auto GEMs from the template model Yeast8 (**c**). All the fitting shown as blue lines was based on a generalized additive model, if not stated in this work.

To characterize the trend of yeast genomic and metabolic evolution, model similarity (Material and Methods) based on the occurrence of reactions from draft GEMs was estimated for any two yeast species. Meanwhile, the time-calibrated phylogenetic tree for 332 yeast species from the study [1] was used to infer the evolutionary distance between any two yeast species. We discovered that the Pearson coefficients between model similarity and evolutionary distance were −0.73 and −0.79 (*p*-value < 2.2 × 10^−16^), respectively, for draft GEMs derived from the RAVEN ”KEGG” and RAVEN ”MetaCyc” procedures (Figure 5), indicating that the model similarity inferred from draft GEMs was significantly negatively correlated to the evolutionary distance. We ask whether the draft GEMs had a unique value in such an analysis, thus, the semi-auto GEMs reconstructed from a simplified template model Yeast8 in our earlier work [17] were employed to conduct a similar analysis. Interestingly, we found that the Pearson coefficient between model similarity and evolutionary distance based on semi-auto GEMs was only −0.45 (*p*-value < 2.2 × 10^−16^), which confirmed that the draft GEMs actually showed more diversity in metabolism from various yeast species. Meanwhile, additional calculations were made to determine the quantitative correlations (represented by the Pearson coefficient) between the model similarity and trait similarity. Interestingly, such a concise analysis showed that the Pearson coefficient based on draft GEMs from the RAVEN “KEGG” procedure (Material and Methods) was largest, while the value was lowest for semi-auto GEMs (Figure 5). Here, we argue that, although the draft GEMs cannot be used to predict the cellular traits directly, they may, nonetheless, serve to reflect the divergence in yeast metabolic evolution.

**Figure 6:**
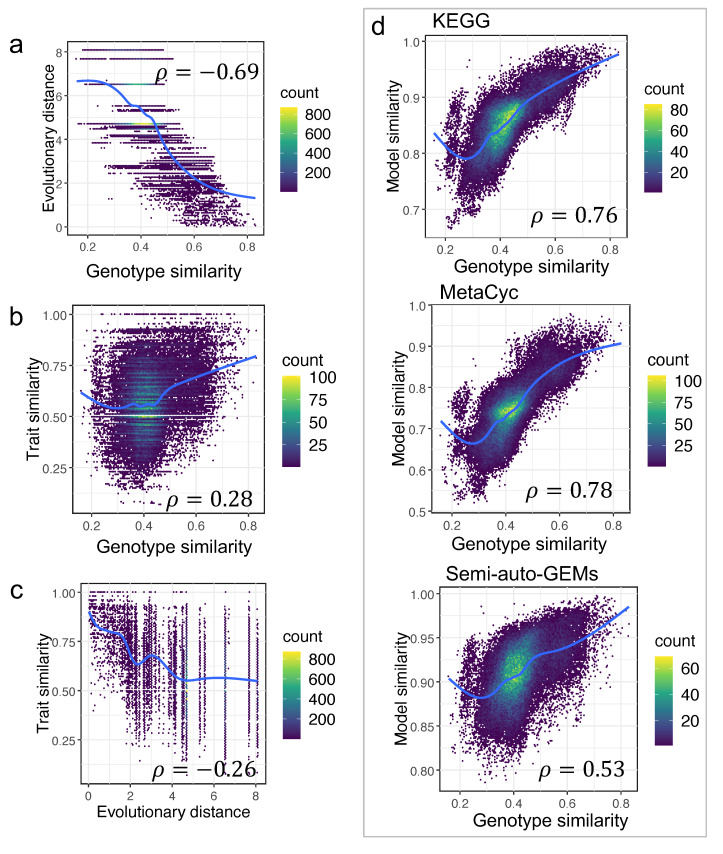
Genotype similarity significantly contributes to model similarity: (**a**) Correlation between genotype similarity and genome evolutionary distance; (**b**) correlation between genotype similarity and trait similarity; (**c**) correlation between evolutionary distance and trait similarity; (**d**) correlation between genotype similarity and model similarity calculated using GEMs of different sources across 332 yeast species.

It shows that various pairings of two yeast species may have a comparable evolutionary distance (Figure 5). As a result, the genotype similarity between any two yeast species was further estimated based on the ortholog gene relationships from the study [1] (Material and Methods). The correlation between evolutionary distances and genotype similarity was initially assessed, and the associated Pearson coefficient was −0.69 (*p*-value < 2.2 × 10^−16^) (Figure 6a). In the above analysis, the Pearson coefficient between model similarity and trait similarity ranged from 0.17 to 0.36, which was a very tiny value. Thus, the correlation between trait similarity and genotype similarity or evolutionary distance was further re-analyzed. We found that neither genotype similarity nor evolutionary distance were well correlated with trait similarity (Figure 6b,c), with the corresponding Pearson coefficients of 0.28 and −0.26 (*p*-value < 2.2 × 10^−16^), respectively. Actually, yeast trait diversity can be influenced both by genotype and growth environment, thus, it makes sense that there are no strong relationships among trait similarity and model similarity, evolutionary distance, or genotype similarity.

We still found that, as compared with the semi-auto GEMs, the model similarity based on draft GEMs from RAVEN was more correlated with genotype similarity as the corresponding Pearson coefficients for RAVEN “KEGG”, RAVEN “MetaCyc”, and “semi-auto GEMs” were 0.76, 0.78, and 0.53 (*p*-value < 2.2 × 10^−16^), respectively (Figure 6d), once again showcasing that draft GEMs have advantages in characterizing yeast genomic evolution.

### 3.5. Model Similarity Comparison between Semi-Auto GEMs and Draft GEMs

**Figure 7:**
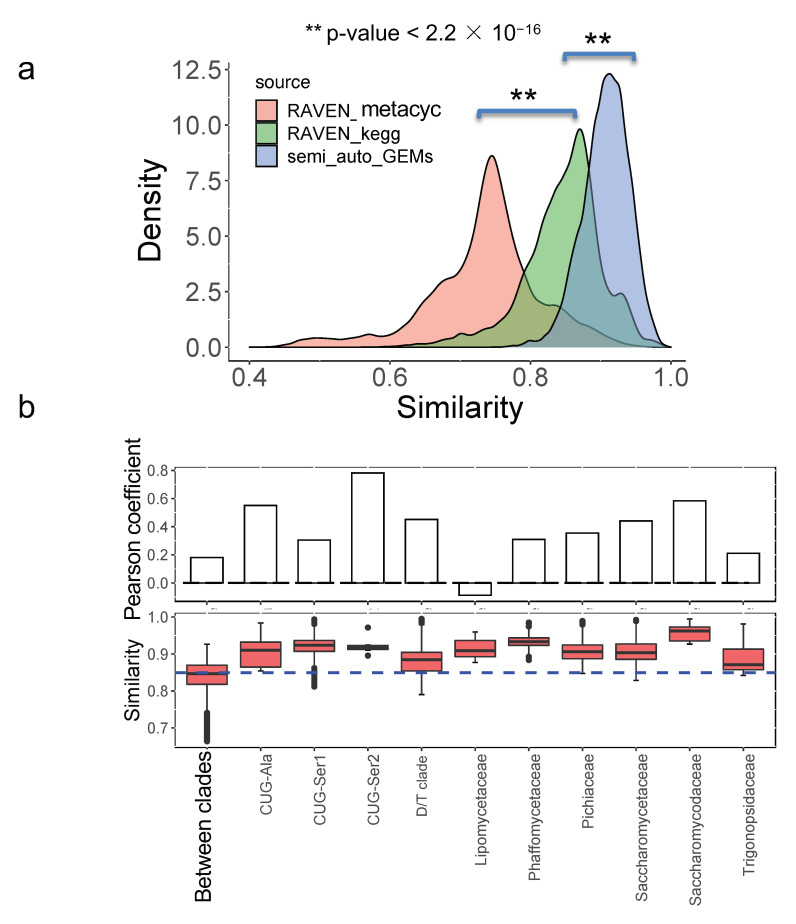
Similarity comparison for metabolic models from different procedures: (**a**) Models from the RAVEN toolbox are more dissimilar from each other than that from semi-auto GEMs from a template model; (**b**) model similarity analysis of main clade level for 332 yeast species based on draft GEMs from the RAVEN “KEGG” procedure. The Pearson coefficient is calculated to represent the correlation between model similarity and trait similarity.

Finally, we further compared the model similarity between draft GEMs and the semi-auto GEMs quantitatively. It initially showed that the model similarity based on semi-auto GEMs was significantly larger than that based on draft GEMs from the RAVEN “KEGG” procedure (*p*-value < 2.2 × 10^−16^), while the least model similarity was based on the RAVEN “MetaCyc” procedure (*p*-value < 2.2 × 10^−16^) (Figure 7a). This suggested that semi-auto GEMs were more similar to each other and that the original draft GEMs without any refinement retained some of the differences in yeast metabolism.

We subsequently assessed the model similarity within or between the major clades for 332 yeast species [1] using draft GEMs from the RAVEN “KEGG” procedure as the corresponding model similarity was better associated with the trait similarity. By comparison, it is shown that the model similarity is substantially higher within the major clades (taking the CUG-Ala as an example, *p*-value = 5.784 × 10^−5^) (Figure 7b) than that across clades, consistent with the analysis from bacteria [30]. The distribution of model similarity among different clades varied noticeably; therefore, the associations between model similarity and trait similarity were further evaluated at the major clade level. We found that, generally, the Pearson coefficient between model similarity and trait similarity was lower for yeast species from different clades than that from the same clade (Figure 7b), suggesting that evolutionarily distant species tended to be divergent in their traits through long-term evolution and adaption. However, we also found that the Pearson coefficient at the major clade “*Lipomycetaceae*” was lowest, which was only −0.08 (*p*-value = 0.6135) (Figure 7b). In contrast to yeast species from the other major clades, it appears that there is no direct positive correlation between model similarity and trait similarity for yeast species from *Lipomycetaceae*, while the related model similarity is in the range of 0.87–0.96 and trait similarity is in the range of 0.40–0.89. When examining the environmental origins of the yeast species from *Lipomycetaceae*, it has been reported that species from this major clade had a widespread distribution [31] and could grow in the soil or in association with insects. Therefore, despite maintaining similar metabolic network structure, due to the close evolutionary distance, we speculated that the distinct growth environments may contribute to the trait variation found in some yeast species.

## 4. Discussion

In this work, we built a pan-draft metabolic model for yeast species based on 332 different draft GEMs to probe the conservatism and diversity underlying yeast genomic and metabolic evolution. The procedure to build the pan-draft model is different from previous studies where the models were built by merging the reaction information from several existing GEMs of typical fungal species [12,18]. It is possible that, in this procedure, only several typical organisms’ metabolic capabilities would be fully represented by this type of pan model, while the metabolic diversity of less-studied organisms would be overlooked to some extent. To overcome this issue, first, we obtained draft GEMs for each yeast species with the aid of the RAVEN toolbox [14], where two comprehensive biochemical databases, KEGG and MetaCyc, were set as the reference to build the species-specific model. Using such a procedure, the metabolic potential and diversity of each yeast species could be fully characterized. From the model similarity analysis, it clearly shows that although the draft GEMs cannot be used to predict the cellular phenotypes directly based on the constraints from genetic or physiological parameters, they are more dissimilar as compared with those from the semi-auto GEMs [17]. It should be noted that, in large-scale GEM reconstruction, the “template model” procedure has been preferred as it guaranteed that the semi-auto GEMs could have the prediction capability conveniently [12,17]. However, some drawbacks may exist with this procedure. One challenge is that the semi-auto GEMs can be similar to each other, therefore, not reflecting the subtle metabolic evolution across different species/strains (Figure 7a). As a result, these semi-auto GEMs may not be the best starting point for the pan-reactome analysis for a group of interesting species. In this respect, draft GEMs and the pan-draft models for yeast species in this work exhibited potential advantages for exploring the general tendencies in yeast metabolic evolution. As stated in this work, according to Heaps’ law, the pan-reactome of yeasts belong to a type of “closeness” in evolution, and the size of the pan-reactome nearly reaches stable once the number of sampled species is over 50 (Figure 3), which, thus, indicates the relatively high conservatism in yeast metabolic evolution during the past 400 million years [1]. We think that this is the first time to illustrate the tendencies in the evolution of the yeast pan-reactome through a large-scale draft GEMs reconstruction and integrative analysis. However, the quality of draft GEMs should be further improved as it could be influenced by multiple factors, such as the algorithms used for gene function annotation and the types of biochemical reaction database [32]. Note that the draft GEMs cannot be used to predict the cellular phenotypes directly, the refinement of semi-auto GEMs based on draft GEMs will be undoubtedly important for the modeling work of non-conventional yeast species.

Estimating the quantitative correlations between genotype and phenotype is always a challenging task in biology studies as so many factors could influence the final cellular traits, such as the auxotroph [33], enzyme promiscuity [34], the lack of transcriptional factor [35], and the reaction gaps in the downstream pathways [17]. Together with the rich genomic and physiological datasets [25], large-scale draft GEMs reconstruction and comparative analyses provide unprecedented chances to explore the correlations among genotype, evolutionary distance, model similarity, and trait similarity. Our analysis initially shows that evolutionary distances and environmental differences could shape the divergence in yeast trait evolution, supporting the view that both intrinsic physiological factors and extrinsic ecological factors drive yeast metabolic diversity [36]. Consistent with the observation that draft GEMs are more dissimilar than semi-auto GEMs for paired yeast species, we found that the model similarity of draft GEMs was more correlated with evolutionary distance and trait similarity, which performs better than semi-auto GEMs (Figure 6). However, the correlation between draft GEMs similarity (genotype similarity) and trait similarity is relatively small, with the largest Pearson coefficient of 0.36. Thus, it initially indicates that the presence of metabolic potential in the genome is not sufficient to predict the phenotype of the yeasts. In our analysis, whether or not the yeast could use different substrates qualitatively was defined as the so-called “trait”. However, there are still many other types of quantitative traits, i.e., the specific growth rate and stress tolerance, which were not considered in our analysis; thus, more standard physiological datasets would be beneficial to fully represent the yeast metabolic diversity and reflect the genomic evolution. It should be noted that the draft GEMs only represent the existence of related reactions based on genome annotation, but how the corresponding enzymes participate in real metabolic activities within cells was not clear, and the advanced modeling together with the multi-omics integrative analysis, will be significant to decipher the complex relations between genotype and phenotype during yeast’s long-term evolution [9].

## 5. Conclusions

Through the reconstruction of draft GEMs and a pan-draft metabolic model for all 332 yeast species, we discovered that the metabolism of yeasts is quite conservative, with the pan-reactome for the existing yeast species belonging to the type of “closeness” through long-term evolution. Additionally, it was demonstrated that draft GEMs directly from RAVEN without any refinement could better characterize the yeast genomic and metabolic diversity as compared with the semi-auto GEMs from a template model. Comparatively, the model similarity calculated by draft GEMs based on the RAVEN ”KEGG” procedure had the highest correlations with trait similarity among yeast species. However, neither model similarity nor genotype or evolutionary distance correlated with trait similarity well (|Pearson coefficient| ≤ 0.36), suggesting that trait diversity may be shaped by multiple factors. Consistently, it shows that diverse growth environments seem to contribute to trait diversity among yeast species from specific evolutionary clades. The pan-metabolic model and draft GEMs from this study should serve as important resources for enhancing the current yeast species-specific GEMs and for shedding light on the intricate relationships among genotype, growth environment, and yeast phenotypic diversity.

## Data Availability

All the draft yeast species models and the related scripts can be downloaded from https://github.com/SysBioChalmers/Yeast-Species-GEMs, accessed on 19 October 2022.
